# Recurrence of a neuroendocrine tumor of adrenal origin: a case report with more than a decade follow-up

**DOI:** 10.1186/s12902-020-00673-7

**Published:** 2021-01-07

**Authors:** Fatemeh Rahmani, Maryam Tohidi, Maryam Dehghani, Behrooz Broumand, Farzad Hadaegh

**Affiliations:** 1 Prevention of Metabolic Disorders Research Center, Research Institute for Endocrine Sciences, Shahid Beheshti University of Medical Sciences, No. 24, Yamen Street, Velenjak, Tehran, Iran; 2 Emeritus Professor of Medicine, Iran University of Medical Sciences, Pars Advanced and Minimally Invasive Manners Research Center, Pars General Hospital, Keshavarz Blvd, Tehran, Iran

**Keywords:** Case report, Neuroendocrine tumor, ACTH-secreting neuroendocrine tumor, Ectopic ACTH syndrome

## Abstract

**Background:**

Neuroendocrine tumor (NET) with adrenocorticotropic hormone (ACTH) secretion are very rare. To our knowledge, no follow-up study is published for ACTH-secreting NET, regardless of the primary site, to show second occurrence of tumor after a long follow-up, following resection of primary tumor.

**Case presentation:**

Here, we describe a 49-year-old-man with cushingoid feature, drowsiness and quadriparesis came to emergency department at December 2005. Laboratory tests revealed hyperglycemia, metabolic alkalosis, severe hypokalemia, and chemical evidence of an ACTH-dependent hypercortisolism as morning serum cortisol of 57 μg /dL without suppression after 8 mg dexamethasone suppression test, serum ACTH level of 256 pg/mL, and urine free cortisol of > 1000 μg /24 h. Imaging showed only bilateral adrenal hyperplasia, without evidence of pituitary adenoma or ectopic ACTH producing tumors. Importantly, other diagnostic tests for differentiating Cushing disease (CD) from ectopic ACTH producing tumor, such as inferior petrosal sinus sampling (IPSS), corticotropin releasing hormone (CRH) stimulation test, octreotide scan or fluorodeoxyglucose (FDG)-positron emission tomography (PET) scan were not available in our country at that time. Therefore, bilateral adrenalectomy was performed that led to clinical and biochemical remission of hypercortisolism and decreased ACTH level to < 50 pg/mL, findings suggestive of a primary focus of NET in adrenal glands. After 11 years uncomplicated follow up, the ACTH level elevated up to 341 pg/mL and re-evaluation showed a 2 cm nodule in the middle lobe of the right lung. Surgical excision of the pulmonary nodule yielded a carcinoid tumor with positive immunostaining for ACTH; leading to decrease in serum ACTH level to 98 pg/mL. Subsequently after 7 months, serum ACHT levels rose again. More investigation showed multiple lung nodules with metastatic bone lesions accompanied by high serum chromogranin level (2062 ng/mL), and the patient managed as a metastatic NET, with bisphosphonate and somatostatin receptor analogues.

**Conclusion:**

This case of surgically-treated NET showing a secondary focus of carcinoid tumor after one decade of disease-free follow-up emphasizes on the importance of long-term follow-up of ACTH-secreting adrenal NET.

## Introduction

Neuroendocrine tumors (NETs) are a family of heterogeneous neoplasms of epithelial or neuronal/neuroectodermal origin, arising from any anatomical site. These neoplasms, express different proteins including markers of general neuroendocrine differentiation and site-specific markers such as hormones and transcription factors [1]. The overall incidence of NET is ~ 2 per 100,000 cases per year [2]. The ectopic adrenocorticotropic hormone (ACTH)-dependent Cushing syndrome (CS), an occasional manifestation of NETs, is caused most commonly by small cell lung carcinomas, following by bronchial carcinoid tumors, thymic carcinoids, islet cell tumor of pancreas, medullary thyroid carcinoma, and rarely pheochromocytoma [2]. However, data regarding the prevalence of ACTH-secreting NETs are limited. In a tertiary referral center, among 918 patients with thoracic and gastroenteropancreatic NETs, the prevalence of ACTH-producing NET was 3.2% [3] .

To the best of our knowledge, this is the first case of ACTH-secreting NET, with improvement of symptoms and signs of hypercortisolism and normalization of the serum ACTH level after bilateral adrenalectomy, with long term of remission, complicated by the recurrence of the tumor in the lung with multiple bone metastasis after 11 years.

## Case presentation

**Fig. 1:**
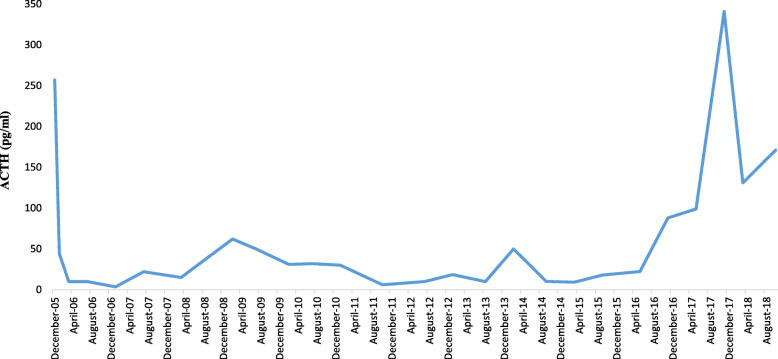
Trend of serum ACTH level from December 2005 to 2020

**Fig. 2:**
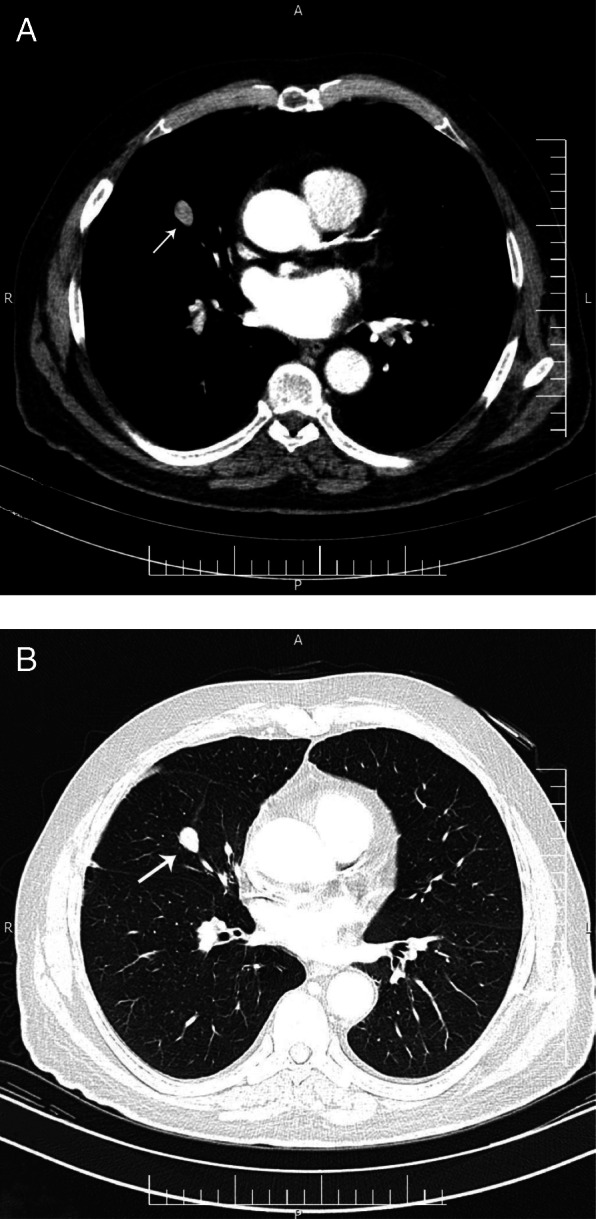
Spiral chest computed tomography of the patient in 2017. **a** mediastinal view, **b** parenchymal view of lung nodule (arrow)

**Fig. 3:**
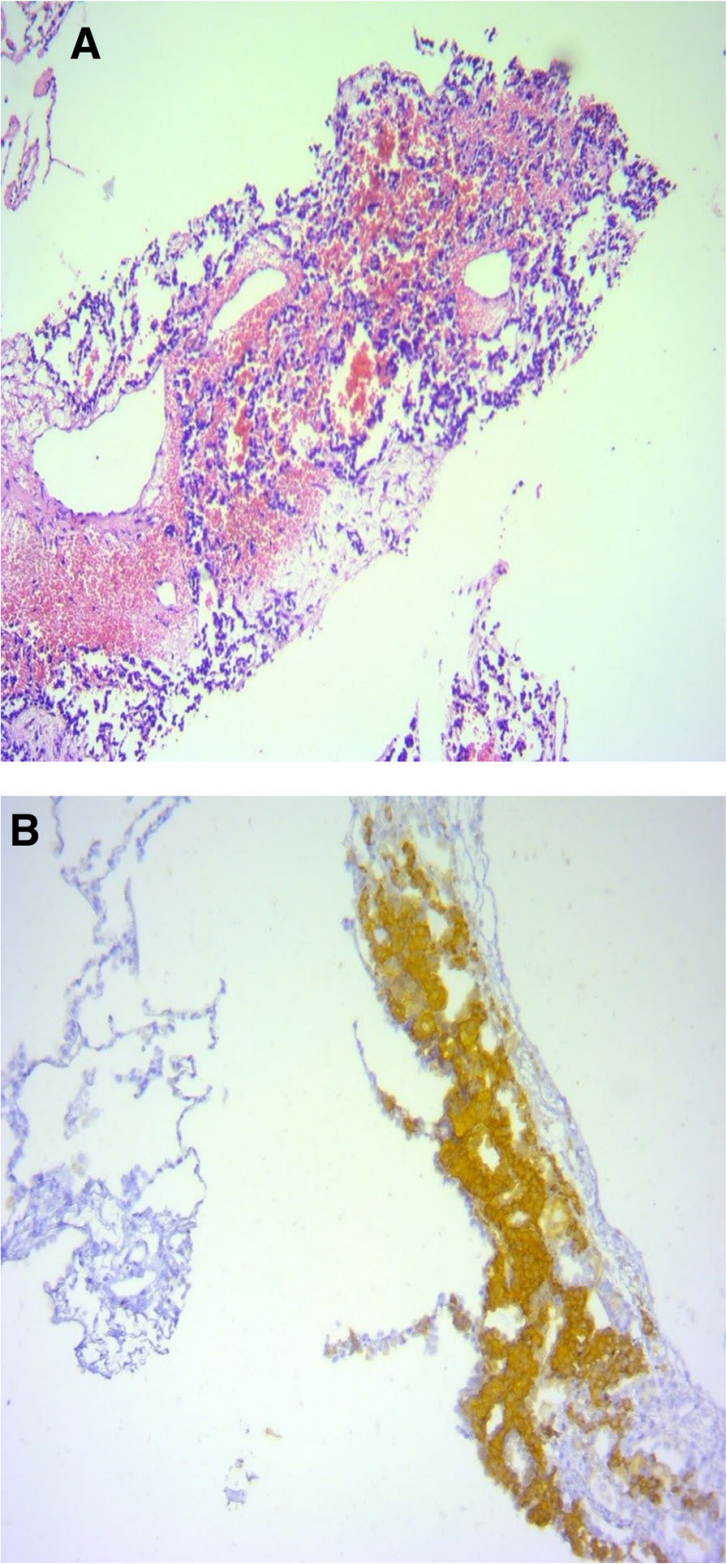
Histopathology of lung nodule biopsy: **a** small monotonous epithelial cells, without atypia or mitotic activity (H&E staining, magnification × 100); **b** strong positive staining of tumor cells for ACTH (magnification × 100)

**Fig. 4:**
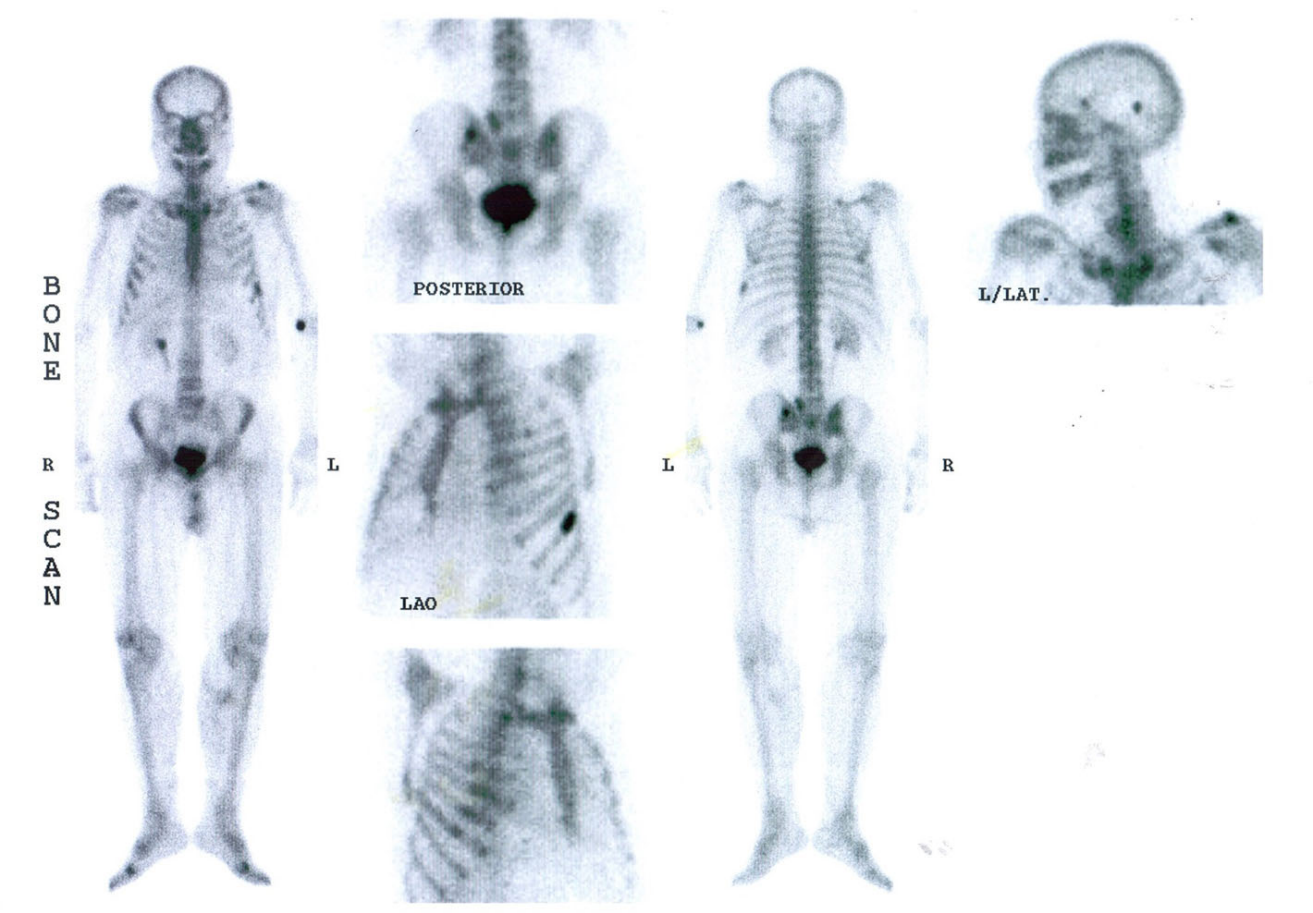
Whole body scan with 99 m technetium. Multiple bone metastases in clavicles, ribs, iliac, temporal, and parietal bone

A 49-y-old man admitted to an emergency department in 21 December 2005, with the complaint of drowsiness and quadriparesia. On admission, the patient was afebrile and had a blood pressure of 200/110 mmHg, pulse rate of 95/min and, a body mass index of 32 kg/m^2^. On physical examination, he had facial plethora, central obesity, pitting edema of limbs, without evidence of purple striae or hyperpigmentation. On neurologic examination, cranial nerves function was intact, proximal and distal force were 3/5, and tendon reflexes were diminished. Other examination was unremarkable. His problem was started a year ago with weight gain (4 kg), fatigue, proximal muscle weakness, easy bruising and hypertension. He didn’t complaint paroxysmal hypertension, headache, palpitation, or sweating. He had been smoker of 20 pack/y for almost 30 years, with no previous medical or surgical history, and no family history for endocrine disease. Initial work up on admission revealed marked hypokalemia (2.1 mEq/L), metabolic alkalosis (pH: 7.58, HCO3:57.2 mEq/L) and blood glucose of 330 mg/dL. Electrocardiogram showed long QT interval (0.52 s), inverted T wave, and prominent U wave in precordial leads. The patient admitted in coronary care unit. According to high suspicious of CS, 24-h urine for urinary free cortisol (UFC) was collected. Laboratory data revealed UFC greater than 1000 μg/24 h (reference value: 50–149 μg/24 h), serum ACTH 257 pg/mL at 8 AM (reference value: 9–46 pg/mL), and morning serum cortisol 57 μg/dL (reference value:5.5–26.1 μg/dL) (Table 1). Following 8 mg oral administration of dexamethasone at 11 PM, no suppression was found at morning serum cortisol level (67 μg/dL). Considering ACTH-dependent CS, dynamic pituitary magnetic resonance imaging (MRI) was done that did not show pituitary adenoma; spiral chest and abdominopelvic computed topographies (CT) were unremarkable, except of the significant enlargement of bilateral adrenal glands. Treatment with ketoconazole, 200 mg every 12 h, was initiated to control hypercortisolism. According to persistent hypokalemia despite excess potassium supplement (> 120 mEq/day) 2 days after starting ketoconazole, the patient was scheduled for bilateral trans-abdominal open adrenalectomy on 28 December 2005. The weights and sizes of excised right and left adrenal glands were 18 g, 6× 3× 0.8 cm and 20 g, 6× 3.5× 1 cm, respectively. Microscopic examination revealed diffuse adrenocortical hyperplasia. Three days after surgery, 24-h UFC, morning serum cortisol and ACTH levels decreased to 27 μg/24-h, 2.2 μg/dL, 44 pg/mL, respectively, furthermore blood pressure and serum potassium and glucose levels were normalized. The patient was discharged on daily dose of 5 mg prednisolone and 0.1 mg fludrocortisone. All signs and symptoms of CS were resolved gradually during 4 months, and 24-h UFC was consistently less than 4 μg/24-h. He remained asymptomatic and during annual laboratory follow-ups results of serum ACTH and UFC were unremarkable, i.e. ACTH < 50 pg/mL, UFC < 4 μg/24-h. In November 2016, serum ACTH began to rise, and in November 2017 reached to 341 pg/mL (Fig. 1). Reassessment for ectopic ACTH producing NET was performed using spiral neck, chest, and abdominopelvic CT-scans. A 2 cm mass in the middle lobe of the right lung was found and dynamic contrast enhanced pituitary MRI and Technetium-99 m-octerotide scan were normal (Fig. 2). A CT-guided biopsy from the lung mass showed a tumor composed of solid nests of small monotonous cells with no atypia or mitotic activity, suggesting an ACTH-producing carcinoid tumor. Histologic examination of the resected right middle lobe revealed carcinoid tumor without involvement of hilar, subcarinal and intralobar lymph nodes. Immunohistochemical (IHC) staining showed diffuse positivity for chromogranin, synaptofysine, and ACTH (Fig. 3); the proliferation marker of Ki-67 was positive in 1% of the neoplastic cells, with the final diagnosis of ACTH-producing carcinoid tumor. Postoperative course was uneventful, and serum ACTH level decreased to less than 100 pg/mL. Approximately 7 months later, serum ACTH level had an upward trend to 171 pg/mL (Fig. 1). Spiral chest CT scan revealed at least 2 nodules measuring up to 5 mm in the lower lobe of the right lung. There were also suspicious lytic bone lesions in thoraco-abdominal CT. Subsequently, whole body bone scan with TC^99^ was performed suggesting multiple metastatic bone lesions at clavicles, ribs, iliac, temporal and parietal bones (Fig. 4). CT-guided left iliac wing biopsy revealed thick sclerotic osteoid tissue, without neoplastic involvement, IHC staining for cytokeratin and chromogranin were negative, although serum chromogranin level was reported 2062 ng/mL (reference value: < 100 ng/mL). Hence, according to the high level of the chromogranin, as well as the presence of nodular lesions in the lung, the patient was managed as a metastatic NET, treatment with bisphosphonate and somatostatin receptor analogous was started.

**Table 1:** Laboratory tests of the patient on the first admission in December 2005

Test	Patient value	Reference range
**Hematology**		
WBC	12,800/mm^3^	4500–11,000/mm^3^
Neutrophils	88%	55–70%
Hemoglobin	13.4 g/dL	13.5–17.5 g/dL
Platelets	248,000 mm^3^	150,000–450,000/mm^3^
**Biochemistry**		
B.U.N.	19 mg/dL	8–20 mg/dL
Creatinine	1.51 mg/dL	0.5–1.5 mg/dL
sodium	144 mEq/L	132–145 mEq/L
potassium	2 mEq/L	3.8–5.6 mEq/L
ALT	36 IU/L	5–40 IU/L
AST	21 IU/L	5–40 IU/L
LDH	857 U/L	250–500 U/L
Fasting glucose	330 mg/dL	70–110 mg/dL
Hb A1C	5.30%	4.1–6.6%
Total cholesterol	177 mg/dL	less than 200 mg/dL
TG	180 mg/dL	50–190 mg/dL
calcium	8.5 mg/dL	8.6–10.6 mg/dL
magnesium	2.5 mg/dL	1.6–3 mg/dL
PH	7.59	7.35–7.45
PCO2	61.1 mmHg	35–45 mmHg
HCO3^−^	57.2 mmol/L	24–28 mmol/L
**Hormonal assay**		
Cortisol 8 a.m.	57 μg/dL	5.5–26.1 μg/dL
Urine free cortisol	1020 μg/24 h	40–145 μg/24 h
ACTH	256 pg/mL	0–50 pg/mL
TSH	0.3 μIU/mL	0.23–4.84 μIU/mL
Total T4	6.3 μg/dL	4.5–12.5 μg/dL
Testosterone	1.2 ng/	2.3–10 ng/mL
LH	0.4 IU/L	0.63–7.89 IU/L
Aldosterone (supine)	94 ng/mL	10–105 ng/mL
Direct Renin	3.8 μIU/mL	0.5–1.9 μIU/mL

*WBC* White blood cells, *BUN* Blood urea nitrogen, *ALT* Alanine aminotransferase, *AST* Aspartate aminotransferase, *LDH* Lactate dehydrogenase, *TG* Triglycerides, *ACTH* Adrenocorticotropic hormone, *TSH* Thyroid stimulating hormone, *LH* Luteinizing hormone

## Discussion

Here, we reported a unique case of ACTH-dependent CS due to ACTH-producing NET of adrenal glands origin that resolved after bilateral adrenalectomy, and the patient was on remission for more than a decade follow-up with unremarkable ACTH level. Then rising serum ACTH level indicating the recurrence of other ectopic ACTH secreting NET. Finally, the presence of second focus of NET in lung accompanied by high level of serum chromogranin and bone metastasis was confirmed.

NETs are uncommon tumors, and due to their insidious presentation and difficulty in localization, the diagnosis and treatment of them are challenging [4]. Traditionally, they categorize from well-differentiated to poorly-differentiated tumors according to their histologic feature, lymphovascular invasion, mitotic activity, ki67 labeling index, presence of metastasis, and their hormonal production. Furthermore, the behavior of NET can change during years from low to high grade and from functional to non-functional or vice versa [5].

Here, we discussed the potential scenario for our case. At presentation, there was a possible small size tumor in adrenal medulla (i.e. a small focus of NET). After bilateral adrenalectomy, a significant decrease in ACTH level occurred from 257 to 44 pg/ml and remained in less than 50 pg/ml for more than a decade (Fig. 1), the issue indicated the source of ACTH production was potentially attributable to the adrenal glands. The absence of NET in the pathology of severely hyperplasic adrenal glands was due to low suspicion for its diagnosis, preparing inadequate sections on gross and microscopic examination of the resected tissue, and limited availability of IHC for ACTH staining in 2005. This ACTH-producing NET in adrenal medulla cured after bilateral adrenalectomy and reappeared as a secondary focus of potentially high-grade NET involving lung with bone metastasis after 11 years. ACTH-production is one of the paraneoplastic manifestation of lung cancers [6]. Actually, small cell lung carcinoma and lung carcinoid are associated with ACTH secretion in a rate of 4.5 and 5%, respectively [7]. In our review of literature, ACTH-secreting pheochromocytoma is very rare and, only 58 cases have been reported till 2018 [8]. Moreover, according to International Agency for Research on Cancer (IARC) and World Health Organization (WHO) expert consensus proposal, poorly differentiated neuroendocrine tumors (neuroendocrine carcinoma) do not occur in the adrenal or in paraganglia [9]. The majority of ACTH-secreting pheochromocytoma involved unilateral adrenal glands, excluding three cases of bilateral pheochromocytoma, in the background of multiple endocrine neoplasia, MEN IIa [8, 10]. In a retrospective study by Henrik et al. from all 164 cases of CS during 10 years, just two of them were ectopic ACTH-secreting tumors arising from adrenal medulla [11]. One of them was a 44 years old woman with established ACTH-dependent CS (serum cortisol: 24.3 μg /dL, ACTH: 44 pg/mL), with a heterogeneous 5 cm large left adrenal mass, underwent left adrenalectomy. The patient didn’t have any symptom or sign of pheochromocytoma except hypertension crisis during surgery. After surgery, the symptoms of hypercortisolism resolved accompanied by normal serum levels of ACTH and cortisol. Adrenal pathology revealed just adrenal medullary hyperplasia and IHC staining expressed ACTH in 10% of the adrenal medullary cells. Actually, in small adrenal mass (less than 1 cm) differentiating pheochromocytoma from adrenal medullary hyperplasia is so difficult [12, 13]. In our case we did not exclude the possibility of adrenal medullary hyperplasia with ACTH producing activity, as well. To our knowledge, no follow-up study is published for ACTH-secreting NET, regardless of the primary site, to show recurrence of tumor after a long-time following resection of primary tumor.

Since, we followed the patient using just serum ACTH level, and imaging study did not perform till elevated ACTH level was detected, the chronological scenario for occurring of lung NET in our case was not cleared. One possibility is that the lung foci of NET were present long time before elevated ACTH level was detected i.e. conversion of non-functional NETs to functioning tumors. There are many types of NETs with different behavior from slowly to quickly growth pattern [8].

Regardless of the mentioned scenario, at present we have a patient with lung NET and bone metastasis, hence the patient underwent treatment by Sandostatin LAR 20 microgram every 20 day.

Important limitations should be considered in our case presentation. In 2005, other diagnostic tests for differentiating CD from ectopic ACTH producing tumor such as inferior petrosal sinus sampling (IPSS), corticotropin releasing hormone (CRH) stimulation test, octreotide scan or fluorodeoxyglucose (FDG)-positron emission tomography (PET) scan were not available in our country. The first limited series of performing IPSS have been reported in Iran in 2020 [14, 15].

## Conclusion

We reported a unique case of ACTH-secreting NET in adrenal with resolving hypercortisolism and normalization of ACTH level after bilateral adrenalectomy and recurrence of the second ACTH producing NET in the lung after more than a decade follow-up. We emphasized the importance of long-term follow-up of ACTH-secreting adrenal NETs that cured with surgery to diagnose secondary focus of NET, whether functional or non-functional.

## Data Availability

Data are available from the corresponding author on reasonable request.

## Abbreviations

ACTH: Adrenocorticotropic hormone
CD: Cushing disease
CRH: Corticotropin releasing hormone
CS: Cushing syndrome
CT: Computed topographies
FDG- PET: Fluorodeoxyglucose -positron emission tomography
IARC: International Agency for Research on Cancer
IHC: Immunohistochemical
IPSS: Inferior petrosal sinus sampling
MRI: Magnetic resonance imaging
NET: Neuroendocrine tumor
UFC: Urinary free cortisol
WHO: World Health Organization
